# Executives’ unethical behaviour with directions for future research

**DOI:** 10.3389/fpsyg.2022.977130

**Published:** 2022-12-07

**Authors:** Renhong Zhu, Xiaowei Li, Qin Liu, Qihao Zhou

**Affiliations:** ^1^School of Business, Sun Yat-sen University, Guangzhou, China; ^2^School of Electronics, Electrical Engineering and Computer Science, Queen’s University Belfast, Belfast, United Kingdom

**Keywords:** executives’ unethical behaviour, literature research, emerging markets, theoretical framework, immoral activity

## Abstract

Executives’ unethical behaviour is a common phenomenon in business practice and a hot topic for academic research, which has a profound negative impact on the healthy development of our economy and society. In the past two decades, several scholars from different disciplines con-ducted theoretical research and practical explorations on the issue of senior executives’ (un)ethical behaviour and achieved certain research results. However, the existing research in this field still has problems, such as a lack of systematic integration of research results, unclear research hotspots and unclear development directions. Thus, the present study through a bibliometric analysis, conducted a content coding of these 428 papers identified from 2000 to 2020, constructed a theoretical framework by inductively identifying the corresponding concepts. By reviewing the progress of existing research topics, this study summarised a research framework of executives’ unethical behaviour from the perspectives of the antecedents, the behaviour itself and the consequences of unethical behaviour. The study further proposed future research trends and recommendations for conducting research on executives’ unethical behaviour under emerging market scenarios. The research results provide new ideas for developing the theory of executives’ unethical behaviour and promote the in-depth development of the research on executives’ unethical behaviour in the context of emerging markets.

## Introduction

With the rapid development of emerging economies like China, more and more scholars turn their attention to the emerging market and focus on the management issues in these economies. As the market regulations of emerging economies are not yet completed and the legal awareness of enterprise is weak, various cases on the violation of regulations have emerged (Meng et al., 2018). The violations exist not only in many small and medium-sized enterprises but also in leading enterprises or listed companies (Li and Chen, 2013). This factor has negatively affected those countries’ economy and become barriers to their development (Yang and Ming, 2017). Considering the serious social costs brought by the enterprises’ violation of regulations, this phenomenon has triggered considerable discussions in both the industry and the academia.

Developed countries have a well-developed market mechanism to restrict managers. On the contrary, emerging countries have not yet established a well-regulated market, so executives’ unethical behaviour in emerging markets has challenged traditional under-standing of business ethical behaviour in academia. Therefore, there are new opportunities for theoretical innovation on the research of unethical behaviour. Executives are key decision-makers in an organisation. They have great control over the company and can determine its strategy and future direction (Walls et al., 2012; Lian et al., 2019a; Zhang et al., 2020). Thus, scholars always take corporate executives as the subject to study how to reduce business regulatory violations. Many valuable research findings have explored how to improve regulatory compliance through the reduction of executives’ unethical behaviour. Current literature reviews have summarised the status of this topic but there is a lack of systematic and integrated analytical framework. There need to be a more comprehensive and in-depth analysis of current research and future directions. Moreover, issues rising from executives’ unethical behaviour in emerging market also deserve investigation, such as whether traditional research findings are applicable to countries in the emerging market group and how executives’ unethical behaviour may be specially manifested in such context. To fill this gap, we conducted a systematic literature review to meet the practical needs in academics and contribute to the theoretic development on executives’ unethical behaviour.

Prior research on executives’ unethical behaviour showed two main trends: the causes and the consequences of executives’ unethical behaviour. In the theoretical analysis of the causes of executives’ unethical behaviour, firstly, individual characteristics are the key that determines whether executives will engage in unethical behaviour. For example, Hegarty and Sims (1978) argued that different demographic characteristics and personal traits, such as gender, stage of moral cognitive development and control points, are significantly associated with executives’ unethical behaviour based on upper echelons theory. Secondly, the environment is also an important reason for the occurrence of executives’ unethical behaviour. For example, Brass et al. (1998) and Vardi (2001) examined the influence of the cultural or institutional environment on executives’ unethical behaviour from the “bad barrel” perspective. They argued that the social or organisational environment in which executives operate, including traditional conventions, moral norms and institutional orientation, is a major factor contributing to executives’ unethical behaviour. Thirdly, the characteristics of the unethical event itself are important factors in developing unethical behaviour. That is, the characteristics of the event, such as the total number of outcomes and the likelihood of effects, are considered key factors that lead executives to engage in unethical behaviour. In exploring the characteristics of the event, Jones (1991) introduced the concept of moral intensity to measure the severity of the ethical issues involved in the behaviour itself. Jones (1991) argued that moral intensity could have a significant impact on the emergence of unethical behaviour.

In the empirical analysis of the consequences of executives’ unethical behaviour, academics discussed the consequences of unethical behaviour based on the more established characteristics of the market for managers in the West. However, most studies mainly explored the impact of unethical events on the organisation. For example, Grant and Visconti (2006) and Tan et al. (2012) argued that, from a short-term performance perspective, unethical behaviour might gain short-term competitive advantages by reducing operational costs. From another aspect, Marcus and Goodman (1991) and (Lian et al., 2019a) argued that from a long-term performance perspective, unethical events could have a negative impact on the sustainability of an organisation in direct and indirect ways. However, few studies have addressed the impact of unethical behaviour on executives themselves. Moreover, it is generally assumed that when uncovered, executives’ unethical behaviour can lead to lower employment opportunities owing to personal reputational damage. However, such punitive mechanisms for executives are often based on a well-developed market for managers. In addition, the effectiveness of such punitive mechanisms in transitional economies needs to be further explored.

The existing literature has made some contribution to understanding the occurrence of research on executives’ unethical behaviour. However, there is still room for further research in at least two aspects because of the different research focuses. First, research in the field of executives’ unethical behaviour has shown certain research focus and integration trends, such as research based on individual executives’ physiological and psychological aspects, and research based on environmental aspects of the cultural or institutional environment. However, because of the wide range of research content and the differences in the research objectives of different scholars, a consistent system and a research framework in this field regarding the disciplinary knowledge base and a summary and refinement of core research issues are still lacking. Moreover, for the research area of executives’ unethical behaviour, prior literature review focus on description of current research status, and lacking discussion about the shortage and future research directions. Secondly, the rapid development of emerging economies, typically China, over the past two decades has led to more and more scholars focussing on the phenomenon of executives’ unethical behaviour in these economies. However, considering the significant differences between emerging economies and developed Western countries in terms of institutions, markets and cultural systems, the conclusions of studies on traditional executives’ unethical behaviour have been challenged. Research on executives’ unethical behaviour in emerging economies is still in the exploratory stage. Moreover, what valuable research questions should be focussed on and how these research questions differ from those in mature Western economic contexts are worth considering further. Thus, through a literature review, this study will identify the disciplinary theoretical foundations of the field of executives’ unethical behaviour and propose an overall analytical framework. Specifically, this study reveals the current research situation in this area from three aspects: the antecedents of unethical behaviour, the unethical events themselves and the consequences of unethical behaviour. Furthermore, this study identifies research gaps and proposes future research directions and related topics in light of the prevalence of imperfect market mechanisms for professional managers in transition economies.

## Integrated conceptual framework for the study of executives’ unethical behaviour

Executives are often key decision makers in a company, who have a say in the company’s strategies and should bear responsibility for the company’s unethical behaviour (Zhang et al., 2016; Meng et al., 2018). Therefore, a company’s unethical behaviour is, in the final analysis, executives’ unethical behaviour. In other words, if we want to find out why a company has unethical behaviour, we should look at why executives decide to act unethically. This paper, following the concept of unethical behaviour defined by previous studies, focus on the unethical behaviour of executives. According to previous studies, researchers identified the following three main related outcomes of behavioural ethics: first, unethical behaviour that violates the society’s accepted moral norms (e.g., lying, cheating, and stealing); second, regular ethical behaviour that meets the most basic moral standards of the society (e.g., honesty, respect for others); and extraordinary ethical behaviour that goes beyond the society’s most basic moral standards (e.g., charitable giving, whistleblowing) (Treviño et al., 2014). This paper discusses the former one outcome with a focus on the important role of executives in the unethical behaviour. In addition, based on the findings of Tenbrunsel and Smith-Crowe (2008) and Li and Chen (2013), this paper assumes that such unethical behaviour does not necessarily have to be intentional. Rather, it may also be unintentional.

### Selection and coding of literature on executives’ unethical behaviour

Based on the research questions of this paper, we adopted the systematic literature review (SLR) methodology to determine the literature scope (Snyder, 2019). In order to bear in mind the overall situation of relevant studies, find out mainstream theories both home and abroad, and understand the unique characteristics of the imperfect market mechanism for professional managers, we collected and analysed studies worldwide and conducted literature review in a systematic way. With “unethical behavior,” “unethical,” “immoral behaviour,” “immoral,” “non-moral behaviour,” “non-moral,” “misconduct,” “moral hazard,” “cheating,” “corruption” as search topics/titles/abstracts/keywords, we looked for papers published from 2000 to 2020. It is worth mentioning that “pro-organisational unethical behaviour,” “violation,” “Rent Seeking,” “self-efficacy,” “transgressive behaviour,” “deviance,” “Moral disengagement” were not used as search topics, because the search results overlap to a large extent with those using such keywords as “unethical behaviour” and “immoral behaviour.” This suggests that keywords like “pro-organisational unethical behaviour” and “violation” are somehow a specific category of unethical behaviour. To ensure that the papers we collected matched with the research topic, we make sure that all papers contain one or more search terms in the title, abstract or keywords.

As for the selection of journals, we found that papers on unethical behaviour were published on a variety of journals. Despite of this, most of the papers were published on core journals in the field of business ethics as well as on top academic journals in the field of management and economics. So, we decided to look for papers published on top international management journals or professional journals in the field of business ethics, including AMJ (Academy of Management Journal), AMR (Academy of Management Review), SMJ (Strategic Management Journal), OBHDP (Organisational Behaviour and Human Decision Process), JBE (Journal of Business Ethics), and BEQ (Business Ethics Quarterly). In addition, market incompleteness that typically prevail in emerging economies may further lead to incidents of unethical behaviour, but few have analysed the impact of such feature on unethical behaviour as they primarily focussed on policy and cultural context of developed Western countries. Therefore, this paper also covered papers published on top Chinese economic and management journals including Management World, Journal of Management Sciences in China, Nankai Management Review, Economic Research Journal, Accounting Research, China Industrial Economics, and Acta Psychologica Sinica to shed light on the direction for studies on unethical behaviour in the emerging market.

**FIGURE 1:**
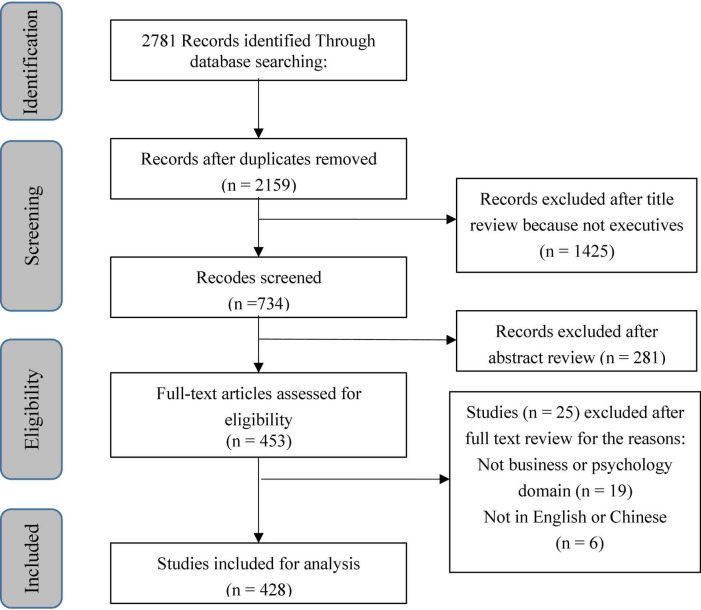
The workflow of systematic literature review (SLR).

**FIGURE 2:**
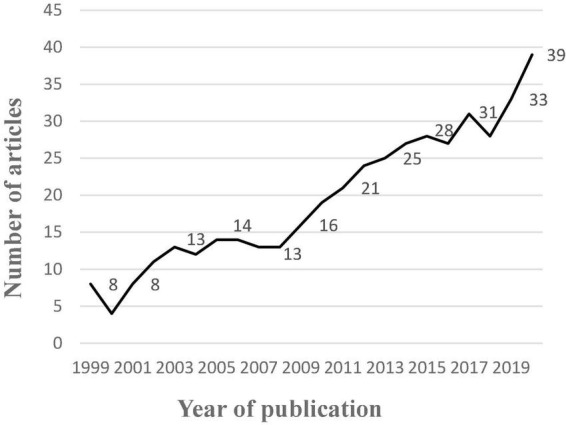
Distribution of literature published over time.

**FIGURE 3:**
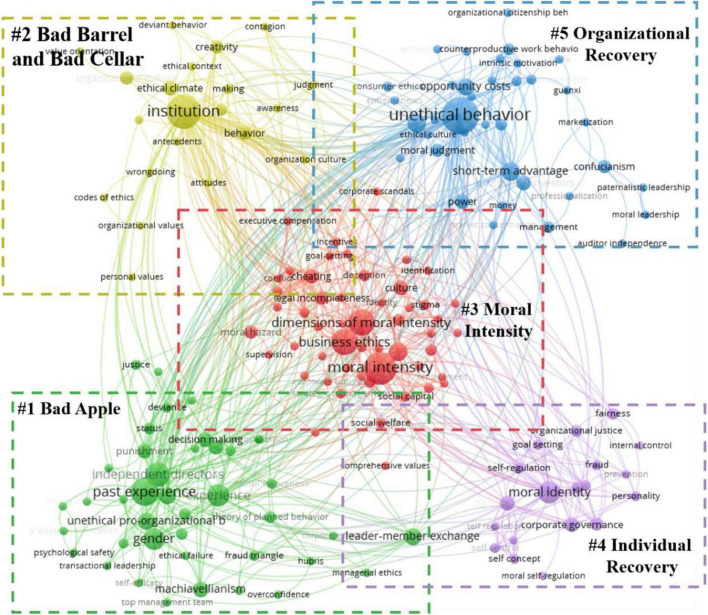
The consequence of co-occurrence analysis.

By searching the literature published in the selected journals using the search terms previously identified, we finally obtained a total of 2,781 documents. And then, we removed the duplicate documents from the search results. However, after removing the duplicates, there were still many documents that did not match the topic of our study. Therefore, to see whether the papers collected were relevant to executives’ unethical behaviour, we carefully read through all the titles and abstracts. Here we used two criteria: (1) the literature must be relevant to the field of business or psychology, and (2) the literature should focus on the important role played by executives in unethical behaviours. After screening, 1,730 articles were removed, and the final sample includes 428 articles. Figure 1 illustrates this work process, and Figure 2 shows the distribution of literature published over time. After identifying these 428 literatures, we then analysed the keywords of these literatures with VOS Viewer software. The co-occurrence analysis results (Figure 3) suggest that five relatively clear keyword clusters emerged among the keywords of these literatures, that is: (1) Bad Apple Phenomenon, which included gender, experience, overconfidence, etc. (2) Bad Barrel Phenomenon, which included organisational culture, Confucianism, Institutional perfection, etc. (3) Moral Intensity, which included moral intensity, magnitude of consequences, dimensions of moral intensity, etc. (4) Individual Recovery Phenomenon, which included social capital, human capital, replacement, etc. (5) Organisational Recovery Phenomenon, which included reputation, Opportunity costs, management changed, etc.

Combined with a panoramic view of the field of executives’ unethical behaviour through a bibliometric analysis, we conducted a content coding of these 428 papers identified and then constructed a theoretical framework by inductively identifying the corresponding concepts. To clarify and reconcile the differences between researchers during the coding process, discussions in depth with researchers were conducted by rereading the content of the literature abstracts during the iterative cycle, to determine the most accurate classification of the literature (Jones et al., 2011; Bettinelli et al., 2017). The coding process is shown in Table 1.

**TABLE 1:** Coding process.

Coding process		
Primary codes	Secondary codes	Research themes
Gender, past experience, education, occupation, nationality, etc.	Executives’ demographics	Bad apple phenomenon
Narcissism, machiavellianism, external or internal locus of control, contagion mechanisms of immorality, etc.	Executives’ psychographics	
Organisational climate, organisational culture, performance pressure, formal ethics institutionalisation, etc.	Organisational environment	Bad barrel and bad cellar phenomenon
Legal incompleteness, cost of legal enforcement, goal orientation, compensation structure, social-level cultural, institutional quality, national welfare level, etc.	Institutional environment	
Jones’ six-dimensional theory, Singhapakdi’s two-dimensional theory, valentine’s single-dimensional theory, etc.	Distinction of moral intensity dimensions	Characteristics of unethical behaviour
Severity of consequences and social consistency, contextual differences, experimental design, etc.	Mechanisms of moral intensity dimensions	
Executive team change, employment opportunities, mechanisms of reputation, penalty level, selection mechanism of executives, etc.	Personal trauma	Individual recovery phenomenon
Social capital accumulation, human capital endowment, “scapegoating” effect, etc.	Career recovery	
Corporate reputation, stakeholder recognition and support, opportunity cost of missing out, short-term competitive advantage, explosive enterprise immoral actions, etc.	Organisational trauma	Organisational recovery phenomenon
Increasing the proportion of independent directors, Increasing the proportion of institutional investors, executive turnover, etc.	Organisational recovery	

### Framework for conceptual analysis of executives’ unethical behaviour

**FIGURE 4:**
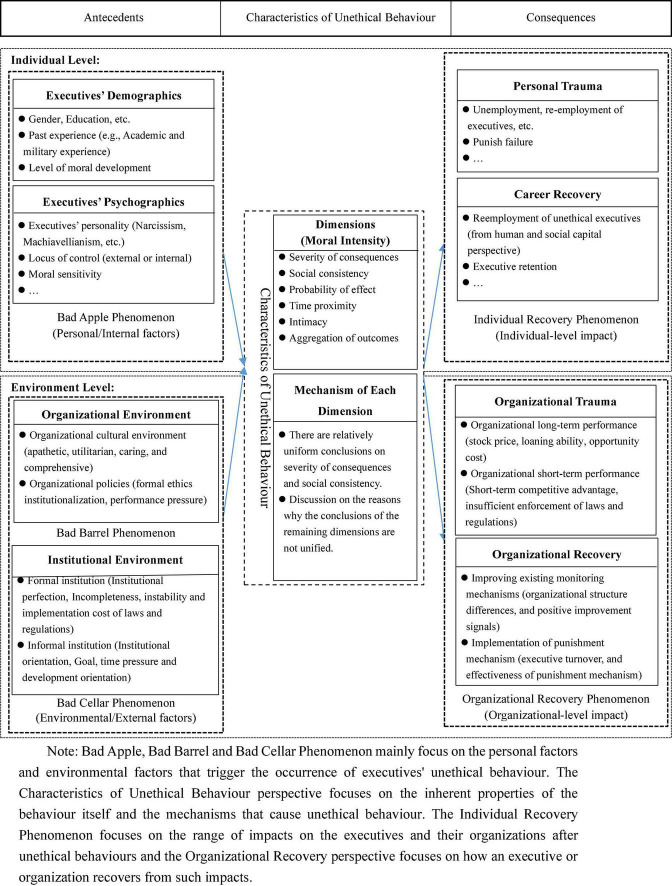
The conceptual framework for executives’ unethical behaviour.

Based on the coding results and the ABC analysis paradigm (Denyer et al., 2008), which is widely used in management and behavioural science research, this study constructs a conceptual analysis framework that can be used to analyse the current research situation in this field, including the antecedents, the characteristics of the behaviour itself and the consequences. For the antecedents, in this study, we focus on the individual characteristics of executives and the environmental factors that trigger and influence the occurrence of executives’ unethical behaviour from the perspectives of bad apple theory and bad barrel theory. The characteristics of unethical behaviour refer to the seriousness of the unethical issues involved in the behaviour, such as the severity and controversial nature of the consequences. Jones (1991) argued that studying executives’ unethical behaviour apart from unethical events is illogical. Thus, the triggers of unethical behaviour cannot be separated from the inherent properties of the ethical issue itself. The consequences of executives’ unethical behaviour refer to the impact of the behaviour on the executives and the organisations. In this study, we call the individual-level impact on the executives as the Individual Recovery Phenomenon. The organisational-level impact is examined from the perspective of ‘organisational recovery’, which is used to describe the trauma and recovery process of the executives’ unethical events on the organisation, just like the trauma and recovery process of biological tissue. However, most of the existing studies focussed on the impact of executives’ unethical behaviour on corporate reputation and stock price. They ignored the process of organisational recovery after the unethical events, that is, how organisations can better recover from the negative impact of executives’ unethical behaviour. Therefore, in the present study, through a review of the prior literature, we proposed a conceptual analysis framework of executives’ unethical behaviour (Figure 4) from three aspects (i.e., the antecedents, the behaviour itself and the consequences) and five dimensions (i.e., bad apple phenomenon, bad barrel and bad cellar phenomenon, characteristics of un-ethical behaviour, Individual Recovery phenomenon and organisational recovery perspective).

We will review papers on the causes of unethical behaviour from the individual level, the organisational level, and the feature of the unethical event itself. Specifically, (1) the bad apple phenomenon refers to physiological and psychological factors that influence executives at the individual level, in which physiological factors include gender (Knight, 2002; Nguyen Nhung et al., 2008), education level (Wang and Zhao, 2007), past academic and military experiences (Franke, 2001; Kish-Gephart et al., 2010; Zeming et al., 2020), etc.; psychological factors include executives’ personality (Donmez-Turan, 2015), locus of control (Detert et al., 2008), etc. (2) Bad barrel phenomenon refers to the influence of institutional and cultural environment at the organisational level, in which cultural environment includes the internal culture of an organisation (Bad Barrel) and the broader social culture of a country (Bad Cellar) (Peterson, 2002; Tan et al., 2012). (3) Different from studies on the bad apple phenomenon or bad barrel phenomenon, studies on the feature of unethical behaviour, such as the severity and controversy, discusses its impact on executives’ unethical behaviour. There are relatively few studies on this topic, and in the limited number of studies that are relevant, researchers mainly explored two constitutive dimensions of moral intensity (Jones, 1991; Tan et al., 2011) and the mechanisms of each dimension (O’Fallon and Butterfield, 2005). In addition, we also reviewed papers on the consequences of unethical behaviour from the individual level and the organisational level. Prior studies mainly explored the impact of unethical behaviour on executives (e.g., reputational damage, turnover, etc.) or organisations (e.g., organisational long- and short-term performance) (Desai et al., 2006; Grant and Visconti, 2006; Karpoff et al., 2008), and how they can recover from such impact.

## Analysis for research themes of previous literature on executives’ unethical behaviour

### Research themes on the antecedents of executives’ unethical behaviour

#### Bad apple phenomenon

The bad apple phenomenon suggests that the executives’ own characteristics, including executives’ demographics and psychographics, are key factors determining whether they will engage in unethical behaviour. Hambrick and Mason (1984) proposed upper echelons theory, which uses executives’ observable demographic variables as proxies for executives’ unobservable and difficult-to-measure psychological processes. Since they proposed this theory, executive demographic variables have received sustained attention from scholars as a central theme in the study of unethical behaviour. These demographic variables include the gender (Andreoli and Lefkowitz, 2009), past experience and education level of the executive. In terms of gender, it is generally believed that female executives have less opportunistic behaviour (Dreber and Johannesson, 2008), greater moral sensitivity (Gilligan, 1977; Liu and Liu, 2014) and a more robust operation approach (Wei et al., 2012) when making corporate decisions because they have a lower propensity for risk and greater consideration of ethical issues (Knight, 2002; Nguyen Nhung et al., 2008). Some scholars have also studied the influence of executives’ past experiences on their unethical behaviour based on identity theory and imprinting theory. For example, the academic experience of executives may reduce the emergence of unethical behaviour, because of accelerating the development of their moral cognition (Wu and Liu, 2006; Wang and Zhao, 2007; Kish-Gephart et al., 2010) and reducing potential reputation crises (Wen et al., 2019; Zeming et al., 2020). However, research findings on executives’ military experience are inconsistent, with some scholars suggesting that military experience fosters greater moral perceptions (Franke, 2001) and greater stress tolerance (Boas et al., 1998), thereby contributing to the reduction of unethical behaviour. On the contrary, other scholars suggested that this case may also lead to a higher willingness to take risks and create overconfidence (Berkowitz and Lepage, 1967; Elder, 1986), leading to executives’ unethical behaviour. The use of executives’ demographic variables as antecedents of unethical behaviour is, in general, a very important subset of the field. However, factors such as gender are only surface-level factors of individual characteristics, so their underlying mechanisms of the influence on unethical behaviour need to be further investigated. In addition, little attention has been paid in the existing literature to the variability of the impact of past experience on executives, such as whether the same experience has different effects at different times of the executive’s life or at different periods of economic development. Therefore, further exploration of the mechanisms underlying the effects of individual demographic variables on executives’ unethical behaviour is a promising research direction.

Apart from executives’ demographic variables, some studies have also explored the antecedents of executives’ unethical behaviour from the perspective of their psychographics, such as executives’ personality and locus of control. Studies on executives’ personalities suggested that executives with ‘dark’ personalities, such as narcissism and Machiavellianism, are significantly and positively associated with unethical behaviour (Donmez-Turan, 2015). Typically, narcissistic executives exhibit traits, such as self-centredness (Morf and Rhodewalt, 2001), disregard for the interests of others (Emmons, 1987) and lower moral sensitivity. From another aspect, high Machiavellian executives are more pragmatic, unscrupulous and utilitarian, and they tend to abandon moral norms to achieve their goals (Wu and Lebreton, 2011). Interestingly, in a study on social media fatigue, Banks et al. (2019) found that social media fatigue predisposes individuals to a lack of motivation and cognitive flexibility, which in turn leads to unethical behaviour, and this effect tends to be more pronounced for female executives (Al-Shatti et al., 2022). This finding contradicts the previous study that male executives have a higher tendency to behave unethically, and therefore also deserves further exploration in future research. Besides, research on locus of control, in addition to executives’ personality, is also of interest to scholars and suggests that individuals with an external locus of control tend to view external forces as determinants of things, and therefore, they tend to have a looser ethical code (Detert et al., 2008). However, compared with the research on executives’ demographic variables, research on psychographic characteristics is still in its infancy and deserves continued attention in future studies.

#### Bad barrel and bad cellar phenomenon

With the development of the study on executives’ unethical behaviour, scholars gradually expanded from the demographic and psychographic characteristics of executives to study the antecedents of their unethical behaviour to the cultural environment and institutional environmental factors, that is, from the perspective of bad apple phenome-non to bad barrel and bad cellar phenomenon.

First, from the perspective of bad barrel phenomenon, current research mainly focuses on the internal environment of the organisation. Organisational environment refers to the values, moral norms and traditional customs shared by members of organisations (Liu and Liu, 2014). Prior literature mainly explored the antecedents of executives’ unethical behaviour from the perspective of organisational-level cultural environments and organisational policies. Based on the different levels of attention enterprises attach to organisational performance and employee well-being, sodm cultural environment can be divided into four types: apathetic, utilitarian, caring and comprehensive type. Amongst them, the apathetic and utilitarian cultural environment neglects attention to the organisation members. In this case, executives tend to have higher motivation for unethical behaviour to maximise the pursuit of the organisation or their own interests (Tan et al., 2012). By contrast, a caring and comprehensive cultural environment will lead to a lower tendency for executives to behave unethically (Deshpande, 1996). Miller et al. (2015) proposed a similar view in their study, where they argued that social comparisons can induce executives to try to restore a kind of “fairness,” which in turn leads to unethical behaviour.

As for organisational policies, Lant (1992) suggests that the pressure on decision makers often comes from the gap between their perceived reality and expectations. When companies set high performance pressures on executives, for example, companies often publicly announce that they are outperforming their competitors and pursuing the “number one” in their mission, executives are more likely to behave unethically. This is because unethical behaviour, while detrimental to stakeholder interests, may pro-vide firms with certain advantages to slow performance declines (Rudy and Johnson, 2016). Moreover, based on the context of COVID-19 pandemic, Agarwal (2021) found that to manage the negative effects of COVID-19 and improve performance, most firms had to make structural and operational changes, such as layoffs and work overload on the remaining employee. In this case it leads to higher job insecurity and excessive stress among executives, which in turn triggers unethical decisions. With the increasing attention to business ethics, companies are also paying more attention to ethical issues in their operations and explicitly include ethical requirements in their policy manuals. As Singhapakdi et al. (1996) has argued, the policies that explicitly include ethical requirements can directly influence the executives’ behaviour and are assimilated in the organisational culture. In addition, it is worth paying attention to the fact that formal ethical norms in organisations have received less attention in studies on emerging economies, so it is worth further exploring whether they can actually reduce executives’ unethical behaviour in emerging economies and the mechanisms of their effects.

Second, from the perspective of bad cellar phenomenon, which focuses on institutional environment at macro-level. The institutional environment has been a hot research topic in recent years in studying the antecedents of executive unethical behaviour, which mainly involves formal institution (institutional perfection, incompleteness, instability and implementation cost of laws and regulations, etc.) and informal institution (institutional orientation, goal, time pressure and development orientation, social cultural environment, etc.) The perfect institutional environment can better protect enterprise property rights and investment returns and reduce transaction costs and risks (Wan and Chen, 2010). This environment can also make enterprises have a strong rule consciousness and more consider the constraints of rules while pursuing economic interests, thereby reducing unethical behaviour. In general, several studies explored the influence of institutional perfection on executives’ unethical behaviour from the perspectives of imperfection, instability and implementation costs of laws and regulations based on institutional theory and institutional anomie theory (He et al., 2015; Jun, 2017).

In addition, some scholars explored the impact of institutional orientation based on existing institutions. Generally speaking, establishing a goal-oriented system will prompt executives to have a higher tendency to behave unethically to achieve the predetermined performance requirements (Kaptein, 2008). By contrast, the development-oriented system will make executives’ pay more attention to the process of goal realisation. This system will also identify the deficiency and potential of organisational members and train them (He et al., 2012), which is negatively correlated with the unethical behaviour of executives. Moreover, scholars have gradually deepened their research on goal orientation. The degree of goal clarity and difficulty, the process of goal setting and the goal content have gradually attracted more and more scholars’ attention. Interestingly, many studies have found that the use of equity incentives for executives may also trigger unethical behaviour (Burns and Kedia, 2006; Efendi et al., 2007; Hass et al., 2016), due to the fact that executives will ignore the manner they achieve the goals, which occurs particularly frequently in emerging markets. As rapid economic development and imperfect institutions are important characteristics of emerging economies (Arnold and Quelch, 1998), at this time, both enterprises and countries are more likely to take ensuring rapid economic growth as their primary goal. Therefore, institutional perfection and institutional orientation are also the elements that should be paid attention to in the research on executives’ unethical behaviour in emerging markets.

As for the social-level cultural environment, prior studies mainly focussed on the influence of several specific social-level cultural environments on executives’ unethical behaviour, such as individualism (Peterson, 2002), universalism (Cullen et al., 2004) and money materialism (Messner and Rosenfeld, 2001). However, prior studies mostly focussed on the western cultural environment and ignored the impact of the high-power distance and collectivism culture that often exists in emerging economies like China. Therefore, future research could explore the impact of different national cultures on executives’ unethical behaviour in emerging markets.

#### Characteristics of unethical behaviour

Different from studies on the antecedents of executive unethical behaviour from the perspectives of bad apple phenomenon or bad barrel phenomenon, studies on the characteristics of unethical behaviour are based on the characteristics of the behaviour itself, such as the severity and controversy of the consequences of the behaviour, to explore their impact on executives’ unethical behaviour. Most of the research in this area is based on the concept of moral intensity introduced by Jones (1991) and considers moral intensity as a multidimensional structure consisting of six dimensions: severity of consequences, social consistency, probability of effect, temporal proximity, intimacy and aggregation of outcomes. This concept is mainly used to measure the severity of the ethical issues involved in the behaviour itself. It is generally believed that the stronger the moral intensity, the lower the likelihood of executives engaging in unethical behaviour (Tan et al., 2011). Depending on different research focuses, studies on moral intensity can be divided into studies on the distinction of moral intensity dimensions and studies on the mechanism of each moral intensity dimension on executives’ unethical behaviour. In terms of distinction of moral intensity dimensions, some scholars used factor analysis to distinguish between two dimensions of moral intensity: social pressure and potential consequences (Singhapakdi et al., 1996). By contrast, others argued that moral intensity can be reduced to a common factor, that is, only one dimension (Valentine and Silver, 2001). However, the six-dimensional structure proposed by Jones has been adopted by many scholars and widely used in subsequent studies.

In terms of the mechanism of each dimension, scholars have come to similar conclusions about the severity of the consequences and social consistency. They generally agree that the more severe the consequences of unethical events or the more socially consistent the judgement of the unethical nature of the behaviour, the less likely decision-makers are to act unethically (O’Fallon and Butterfield, 2005). However, the conclusions of the remaining four dimensions are not uniform, and even diametrically opposed conclusions emerge from the different studies. The reasons for this inconsistency are manifold. McMahon and Harvey (2007), through two groups with different experimental designs, and Janet and Anusorn, through a comparison of studies in Thai and US contexts, suggested that differences in within- and between-group designs, including cultural differences, are the main reasons for the significant differences in research findings (Svetla and Isabelle, 2005). Overall, owing to the large differences in culture and legal systems in emerging market countries, the same ethical issue can bring about different perceptions of individual ethical problems in different regions, that is, the same ethical event may bring about different moral intensities in emerging market countries. Thus, further research on moral intensity based on emerging market contexts would be beneficial to enrich the literature in this area and generate opportunities for theoretical innovation.

### Research themes on the consequences of executives’ unethical behaviour

Different from the previous three perspectives on the antecedents of executives’ un-ethical behaviour, both the individual recovery phenomenon and organisational recovery phenomenon focus on the effects of unethical behaviour from the perspective of the con-sequences of unethical behaviour.

#### Individual recovery phenomenon

Individual recovery phenomenon focuses on the effects of unethical behaviour on executive. Previous studies mainly explored the impact of unethical behaviour on executives in terms of personal trauma and career recovery. Personal trauma, one of the important outcomes of unethical behaviour, refers to a range of effects on the executives, such as unemployment, fines and market exclusion, when their behaviour is uncovered. Studies conventionally explored the issue of executive turnover and re-employment following the occurrence of unethical behaviour based on reputation theory. These studies suggested that the disclosure of unethical behaviour has a negative impact on the reputation of executives in the market for professional managers, which in turn leads to higher turnover rates and lower re-employment opportunities (Arthaud-Day et al., 2006; Desai et al., 2006; Karpoff et al., 2008). However, in some emerging economies, institutional imperfection and a low degree of marketisation in the selection mechanism of corporate executive teams (Weihua, 2011) have made it difficult for the reputation mechanism to function. In addition, the penalties for executives’ unethical behaviour are weak (Li and Chen, 2013). This phenomenon has, to some extent, encouraged executives in emerging economies to engage in unethical behaviour. Therefore, directly applying the same views of Western scholars to research the consequences of executives’ unethical behaviour in emerging economies is difficult. Future research should consider the characteristics of emerging economies and explore how the consequences of unethical behaviour in emerging economies affect the professional development of executives and the effectiveness of penalties for unethical behaviour.

Career recovery refers to a series of recovery measures that executives may take to compensate for the negative impact of their uncovered unethical behaviour. It is estimated that 93% of executives will be removed as a result of the disclosure of the unethical event (Agrawal and Cooper, 2017). Moreover, the subsequent re-employment of those executives whose reputation in the market for professional managers has been damaged has become a hot topic in career recovery research. Career recovery focuses on the social and human capital of those unethical executives and its impact on their re-employment (Gowan and Lepak, 2007). It is generally believed that executives with high social and human capital can improve corporate competence, organisation management ability and corporate relational capital (Coffie and Yeboah, 2018), thereby alleviating the negative effects of reputation damage and improving re-employment opportunities. However, there is a relative paucity of research on career recovery in general, with even less research focussing on emerging economies. In Western research, executive turnover has traditionally meant leaving the company, so previous studies mainly explored the issue of career recovery for unethical executives on this basis. Owing to the non-market-based nature of the executive selection mechanism and the strong bureaucratic overtones, unethical executives in emerging economies may simply cease to serve as the chairman or general manager of a listed company but remain in other positions, such as the deputy general manager or director (Weihua, 2011), and maintain the same salary level. Therefore, future research on these differences in performance could not only test the effectiveness of the current regulatory regime but also provide new research objects and opportunities for theoretical innovation in the field of research on the consequences of executives’ unethical behaviour.

#### Organisational recovery phenomenon

Similar to the Individual Recovery phenomenon, the organisational recovery phenomenon also focuses on the consequences of executives’ unethical behaviour and mainly explores the impact on the organisation from the perspectives of organisational trauma and recovery. The study on organisational trauma is relatively rich, and it is generally believed that executives’ unethical behaviour has a negative impact on organisational performance in the long term (Grant and Visconti, 2006), but probably positively affects the short-term performance of the firm (Tan et al., 2012). Research on long-term performance has been conducted mainly in terms of lower corporate share prices (Agrawal and Chadha, 2005), poorer loaning capacity (Huang, 2013), high penalty costs (Alexander, 1999), opportunity costs of missing out (Griffin, 2003), corporate reputation, organisational legitimacy, external stakeholder identification and support for the organisation (Lian et al., 2019b). Compared with the short-term performance, there are more consistent conclusions on the long-term performance that executives’ unethical behaviour directly or indirectly hinders the sustainable development of the firm. There are different conclusions in the impact of executives’ unethical behaviour on short-term performance. The main reason for the disagreement is that unethical behaviour usually is the decision after weighting the costs and benefits (Fei et al., 2016). Thus, firms may compress corporate costs in the short term because of the implementation of unethical behaviour, and then gain a short-term competitive advantage (Grant and Visconti, 2006; Tan et al., 2012). Emerging economies are often characterised by weak penalties (Jiang and Kim, 2015) and poor implementation of laws and regulations (Henisz and Delios, 2003). Therefore, the negative effects of executives’ unethical behaviour may take longer to become apparent in the firm. Therefore, the empirical evidence and improvement of existing conclusions on executives’ unethical behaviour in the emerging markets context are the focus of attention at this stage.

Compared with organisational trauma, research on how organisations can alleviate or restore the ongoing negative effects of executives’ unethical behaviour, that is, organisational recovery, is less. Previous studies explored this issue mainly in terms of improving existing monitoring mechanisms and implementing penalty mechanisms. Amongst the studies on the improvement of monitoring mechanisms, researchers mainly focussed on the dichotomous relationship of ‘corporate structure-executives’ unethical behaviour’. They studied the effect of the differences in corporate structure on organisational recovery, such as increasing the proportion of independent directors (Burns and Kedia, 2006; Ashbaugh-Skaife et al., 2008) and increasing the shareholding of institutional investors (Li and Li, 2008). Moreover, some scholars noted that the change in the organisational structure of the company not only enhances the effect of the organisational monitoring mechanism but also sends a signal to the public that the organisation is committed to changing the *status quo*, which in turn mitigates the negative impact of the executive’s unethical behaviour (Lian et al., 2019a). In addition, the implementation of penalty mechanisms is also an important way of organisational recovery, which mainly emphasises the behaviour of executives and their corresponding responsibilities. As executives are often considered the spokesperson of the company (Hambrick and Mason, 1984), the choice of replacing an executive after his or her unethical behaviour is exposed can effectively send a signal to the public that the organisation has ‘changed radically’. It benefits not only to quickly restore the company’s image but also to divert the attention of the public and directly reduce the negative impact of the unethical behaviour (Cao, 2015; Xuewen and Fan, 2021). Although sometimes these punishments are totally unjustified for some executives, those executives are often treated by companies as scapegoats to alleviate the effects of unethical behaviour (Brown, 1982; Walsh and Seward, 1990). This finding also explains, to some extent, why 93% of unethical executives are replaced as a result of unethical events. However, in the existing research on organisational recovery, there is a lack of research to test the effectiveness of these measures and the possibility of repeated unethical events in organisations. Particularly in emerging economies, several companies have experienced high-level executives’ unethical behaviour, and a number of industry leaders and listed companies experienced such events (Li and Chen, 2013). Thus, helping such companies recover quickly from unethical events, operate normally in the market and reduce the potential for repeat unethical behaviour is vital to the health of emerging economies.

From the review of executives’ unethical behaviour research themes from 2000 to 2020, we draw the following three findings. Firstly, research on antecedents still focuses on executives’ demographic characteristics and institutional environmental factors. Researchers have analysed the drivers of executives’ unethical behaviour from different theories and perspectives. In addition, there has been an increase in the attention paid to the psychographic characteristics of executives, cultural environment factors and the characteristics of unethical events. However, studies based on the uniqueness of emerging economies are relatively few, which is not conducive to theoretical expansion. Secondly, trauma has always been the focus of research on the consequences of unethical behaviour, of which there has been relatively considerable research on organisational trauma but only a small amount of research on personal trauma of executives. Moreover, how organisations or executives ‘recover’ from unethical events is becoming a hot topic. Given the differences between emerging market contexts and traditional research contexts, exploring the nature of these differences and executives’ unethical behaviour in emerging economies is also a promising direction for future research.

## Directions for future research

With the development of the research on executives’ unethical behaviour, more and more scholars are paying attention to the research on executives’ unethical behaviour in emerging economies. The uniqueness of emerging market contexts has led to several unique issues, and thus, there is a great opportunity for theoretical innovation. The unique characteristics of emerging markets include cultural differences, institutional differences and differences in economic development stages. In terms of cultural differences, high power distance, collectivism and relationship utilisation have received more attention from scholars (Jin et al., 2005; Yiu et al., 2014). In terms of institutional differences, emerging economies, such as China, are still in a period of economic transition, and the typical characteristics of the nature of institutions are dynamic, unstable and imperfect (Peng, 2000; Meyer, 2001). Under this context, the weak implementation of laws and regulations and government intervention are also factors to be considered. In terms of differences in economic development stages, many scholars have highlighted the uniqueness of increased market competition and the rapid industrialisation process in emerging economies (Zhou et al., 2005). On this basis, the unique factors that influence the antecedents and consequences of executives’ unethical behaviour in the emerging market context should be explored. Therefore, future research on executives’ unethical behaviour should be further explored with a focus on emerging markets, and the following are several future research suggestions.

### Antecedents of executives’ unethical behaviour in the context of emerging economies

Future research on the bad apple phenomenon should continue to explore the demographic and psychographic characteristics of executives. Firstly, from the perspective of executives’ demographic characteristics, owing to the rapid economic development of emerging economies, executives in these countries have experienced a significant increase in per capita income and career development opportunities. In addition, more and more specialised types of executives, such as those from humble backgrounds, those with overseas study experience and those with foreign employment experience, are appointed as corporate executives, providing potential new research groups for the research on the antecedents of executives’ unethical behaviour. Moreover, previous studies have identified differences in unethical decision-making of executives with similar experiences. However, they have not explored the underlying drivers of these differences and whether they are influenced by the rapid industrialisation process in emerging economies. Furthermore, the changing environment brought about by economic development can have a significant impact on executives’ personal psychological needs, such as a greater emphasis on personal fulfilment and work-life balance. Therefore, future research could conduct further investigation in the following directions: (1) to explore the differences in the mindset of executives with different experiences in their decision-making and the core mechanisms that drive their unethical behaviour; (2) to explore why executives with similar experiences have different tendencies to behave unethically and the impact of the rapid industrialisation process; (3) to explore the impact of different types of psychological needs on executive decision-making preferences. For example, executives who are more focussed on the realisation of personal values may be more likely to neglect the process of realisation, that is, they are less likely to take ethical considerations into account.

Future research on bad barrel phenomenon should further explore the antecedents of executives’ unethical behaviour in terms of the cultural and institutional environments. For the cultural environment, prior studies mostly focussed on the influence of environmental factors on executives’ unethical behaviour from the perspectives of corporate culture and ethical climate. These studies partly ignored the influence of the more general environment in which executives live, that is, the social-level cultural environment. Thus, future research could explore the factors that contribute to executives’ unethical behaviour more in terms of national culture, such as face culture, relationship culture, high power distance, collectivism and long-term orientation. In addition, as emerging economies open up, their traditional cultures are inevitably impacted by foreign cultures. In this context, the question of how face and relationship culture transform and influence executives’ unethical behaviour in the context is also a special research topic. From an institutional environment perspective, most existing research on institutional inadequacies focussed on the lack of a mature formal system and the instability of the system, with less attention paid to the impact of weak implementation of laws and regulations and weak penalty levels. Moreover, in the emerging market context, the state or the government, in the pursuit of rapid economic development, may emphasise results over process in the policy-making process. This case in turn may affect executives’ unethical behaviour and provide new perspectives and theoretical innovation opportunities for research on the antecedents. Therefore, more attention should be paid to institutional orientation research in the future.

Future research on the characteristics of unethical behaviour can try to expand the underlying theories related to the characteristics of the existing unethical events, or even construct new theories. As mentioned in the previous section, moral intensity emphasises an objective description of the behaviour itself, which implies that the same event should have the same moral intensity in different regions. However, owing to the institutional incompleteness of emerging economies, executives in those economies may have different subjective perceptions of the same unethical event. For example, the occurrence of a food safety incident in a country with a well-developed market system may lead to high fines or even criminal liability. On the contrary, in an emerging economy, executives may be more likely to lead to lower expectations of the severity of consequences because of the lack of relevant laws and regulations. In addition, the current literature has relatively uniform conclusions on the two dimensions of moral intensity, namely, the severity of consequences and social consistency. However, the extent and mechanism of the impact of different damages brought about by unethical events, such as physical damage, economic damage and psychological damage, on executives’ unethical behaviour still need to be further explored. Thus, future research can draw attention to the following issues: (1) in the context of inadequate market systems and imperfect institutions, the perceived moral intensity of executives may be the key influencing factor in determining whether they will engage in unethical behaviour. (2) With regard to the various dimensions of moral intensity, the mechanism of each dimension’s effect on executives’ unethical behaviour can be further refined.

### Consequences of executives’ unethical behaviour in the context of emerging economies

Future research on the Individual Recovery phenomenon should further explore the impact of executives’ unethical behaviour on the executives themselves and how they can recover from it. In terms of personal trauma, traditional governance approaches based on reputation theory are difficult to achieve the desired results in emerging economies because of the unique attribute of less marketized professional managers. It not only changes the existing predictions of reputation theory but may also even have new phenomena that are difficult to explain in the traditional literature. In this context, investigating the impact of different incentives or sanctions on executives themselves, how reputation mechanisms work in emerging market contexts, and the mechanisms by which reputation mechanisms work for different types of executives is instructive. In terms of research on career recovery for executives, the current literature paid less attention to this area in general. As executives can become scapegoats in unethical events, the question of how to help innocent executives overcome the negative effects of unethical events, whether companies will compensate executives who become scapegoats and how executives themselves can avoid becoming scapegoats in unethical events are all important issues for future research. Further research on these phenomena will not only explain some of the particular problems in emerging markets but also help to improve existing theories of executives’ unethical behaviour.

Future research on organisational recovery could further explore the impact of emerging market characteristics on the conclusions of traditional organisational trauma research and the effectiveness of recovery measures. From the perspective of organisational trauma, emerging markets are characterised not only by low levels of the penalty and weak implementation of punitive measures compared with mature Western economies (Henisz and Delios, 2003; Jiang and Kim, 2015) but also by a tendency of group behaviour, which leads to the phenomenon that ‘the law does not punish numerous offenders’ (Li and Chen, 2013). These characteristics and phenomena challenge the underlying assumptions of traditional organisational trauma research and, in turn, have an impact on traditional research conclusions. Thus, future research could focus on the following directions: (1) how emerging market characteristics change the underlying theories of existing organisational trauma research, and how they affect traditional research conclusions. For example, how the phenomenon of ‘the law does not punish numerous offenders’ resulting from group unethical behaviour can develop the organisational legitimacy theory. (2) In emerging economies where the trauma of unethical behaviour may be far less than the benefits to the organisation, exploring how governments or markets can increase the costs of corporate unethical behaviour is an important topic for the future, given the characteristics of emerging markets. In addition, there is a growing interest in organisational recovery in the existing literature. Therefore, future research could investigate the effectiveness of these measures empirically or theoretically and further explore other recovery mechanisms. As unethical behaviour is often a trade-off between pros and cons (Fei et al., 2016), follow-up studies on unethical firms (e.g., whether there is repeated unethical behaviour by firms) are not only a test of the effectiveness of organisational recovery measures but also provide a reference for future policy formulation in a broader sense, which are worthy of continued exploration in future research.

## Conclusions

### Research findings and contributions

By systematically reviewing 428 papers on executives’ unethical behaviour published in top Chinese and international journals between 2000 and 2020, this study constructs a conceptual framework for the analysis of executives’ unethical behaviour. The study also proposes suggestions for future research in the context of emerging markets. Some important trends in the research on executives’ unethical behaviour can be reflected in this study. Firstly, based on the Western economic context, the research on executives’ unethical behaviour has become more mature, and a research map is formed based on the antecedents, the characteristics of the behaviour itself and the consequences. Relatively few studies have focussed on the consequences of executives’ unethical behaviour, particularly on the personal impact on executives. However, the existing research themes are generally rich enough to explain the phenomenon of executives’ unethical behaviour in the Western economic context, both in terms of breadth and depth, including richness and complexity. Secondly, research on executives’ unethical behaviour based on the characteristics of emerging economies has only just emerged, requiring further development of relevant research or theory. As the institutions and cultures of emerging economies are quite different from those of developed Western countries, grasping the essence of such differences is important to draw out scientific research questions on executives’ unethical behaviour and to develop theories based on the characteristics of emerging markets. Thirdly, the research on executives’ unethical behaviour has shown strong practical implications. Issues such as the problem of explosive corporate unethical behaviour brought about by food safety and the rent-seeking behaviour to improve corporate competitiveness have attracted the attention of a large number of scholars, providing a worthwhile theoretical basis for the design of new institutions and policies.

This review has three main contributions. Firstly, the study proposes a complete conceptual framework and conducts an in-depth and systematic literature review. Unlike the existing review articles, which focussed on the antecedents of executive misconduct, this study analyses and summarises the research content and themes of the literature on executives’ unethical behaviour in the past two decades. This study also constructs a research map based on the antecedents, the characteristics of the behaviour itself and the consequences, providing a summary of the current status and progress of existing research in this area. Secondly, this study expands and extends the research map of executives’ unethical behaviour. On the one hand, through content analysis of the existing literature, this study presents for the first time two perspectives on the Individual Recovery and organisational recovery phenomena, which are also the focus of current research. On the other hand, by focussing on the characteristics of emerging market contexts, this study attempts to integrate local research with traditional theory. This not only helps to guide more scholars to focus on the consequences of unethical behaviour and to construct corresponding theories but also promotes the deepening of the field of executives’ unethical behaviour research and further extends the traditional research landscape. Thirdly, the study further subdivides the traditional perspective of bad barrel phenomenon into the cultural or institutional environment, making the research findings more detailed and richer.

### Limitations and future research directions

For sample selection, this study focuses on the papers published on both the international and Chinese management journals or professional journals of business ethics. The study retrieves 428 papers on executives’ unethical behaviour as the object of analysis. Although this sample selection method is one of the most common sample selection methods at present, further refinement of the sample under the condition of sufficient time and funding may make the analysis results more scientific and rigorous. In addition, in terms of time frame, this study includes the literature from 2000 to 2020. While sorting out the entire development process of research on executives’ unethical behaviour, future re-search can also explore the dynamic changes in the focus of research, not only by updating the data over time but also by dividing it into different stages.

## Author contributions

RZ and XL contributed to conception and design of the study. XL, QL, and QZ organised the database and performed the statistical. XL and QZ wrote the first draft of the manuscript. All authors contributed to manuscript revision, read, and approved the submitted version.

## References

[B1] Agarwal P. (2021). Shattered but smiling: Human resource management and the wellbeing of hotel employees during COVID-19. *Int. J. Hosp. Manag.* 93:102765. 10.1016/j.ijhm.2020.102765 PMC9998170 36919177

[B2] Agrawal A. Chadha S. (2005). Corporate governance and accounting scandals. *J. Law Econ.* 48 371–406. 10.1086/430808

[B3] Agrawal A. Cooper T. (2017). Corporate governance consequences of accounting scandals: Evidence from top management, CFO and auditor turnover. *Q. J. Finance* 7:1. 10.1142/S2010139216500142

[B4] Alexander C. R. (1999). On the nature of the reputational penalty for corporate crime: Evidence. *J. Law Econ.* 42 489–526. 10.1086/467433

[B5] Al-Shatti E. Ohana M. Odou P. Zaitouni M. (2022). Impression management on instagram and unethical behavior: The role of gender and social media fatigue. *Int. J. Environ. Res. Public Health* 19:9808. 10.3390/ijerph19169808 36011435 PMC9408035

[B6] Andreoli N. Lefkowitz J. (2009). Individual and organizational antecedents of misconduct in organizations. *J. Bus. Ethics* 85 309–332. 10.1007/s10551-008-9772-6

[B7] Arnold D. J. Quelch J. A. (1998). New strategies in emerging markets. *Sloan Manag. Rev.* 40 7–20.

[B8] Arthaud-Day M. L. Certo S. T. Dalton C. M. D. Dalton D. R. (2006). A changing of the guard: Executive and director turnover following corporate financial restatements. *Acad. Manag. J.* 49 1119–1136. 10.5465/amj.2006.23478165

[B9] Ashbaugh-Skaife H. Collins D. W. Kinney W. R. Lafond R. (2008). The effect of SOX internal control deficiencies and their remediation on accrual quality. *Account. Rev.* 83 217–250. 10.2308/accr.2008.83.1.217

[B10] Banks S. Landon L. B. Dorrian J. Waggoner L. B. Centofanti S. A. Roma P. G. (2019). Effects of fatigue on teamsand their role in 24/7 operations. *Sleep Med. Rev.* 48:101216. 10.1016/j.smrv.2019.101216 31630015

[B11] Berkowitz L. Lepage A. (1967). Weapons as aggression eliciting stimuli. *J. Pers. Soc. Psychol.* 7 202–207. 10.1037/h0025008

[B12] Bettinelli C. Sciascia S. Randerson K. Fayolle A. (2017). Researching entrepreneurship in family firms. *J. Small Bus. Manag.* 55 506–529. 10.1111/jsbm.12347

[B13] Boas S. Eliav Z. Esther B. Micha P. (1998). Correlates of charismatic leader behavior in military units: Subordinates’ Attitudes, Unit Characteristics, and Superiors’ appraisals of leader performance. *Acad. Manag. J.* 41 387–409. 10.2307/257080

[B14] Brass D. J. Butterfield K. D. Skaggs B. C. (1998). Relationships and unethical behavior: A social network perspective. *Acad. Manag. Rev.* 23 14–31. 10.2307/259097

[B15] Brown M. C. (1982). Administrative succession and organizational performance: The succession effect. *Adm. Sci. Q.* 27 1–16. 10.2307/2392543

[B16] Burns N. Kedia S. (2006). The impact of performance-based compensation on misreporting. *J. Financ. Econ.* 79 35–67. 10.1016/j.jfineco.2004.12.003

[B17] Cao X. (2015). The effects of senior executives on turnaround: A literature review. *Nanjing J. Soc. Sci*. 09, 38–45.

[B18] Coffie R. B. Yeboah E. H. (2018). Does the social and human capital of retrenched bankers matter in their reemployment? *J. Hum. Resour. Manag.* 21 14–27.

[B19] Cullen J. B. Parboteeah K. P. Hoegl M. (2004). Cross-national differences in managers’ Willingness to justify ethically suspect behaviors: A test of institutional anomie theory. *Acad. Manag. J.* 47 411–421. 10.2307/20159590

[B20] Denyer D. Tranfield D. Van Aken J. E. (2008). Developing design propositions through research synthesis. *Organ. Stud.* 29 393–413. 10.1177/0170840607088020

[B21] Desai H. Hogan C. E. Wilkins M. S. (2006). The reputational penalty for aggressive accounting: Earnings restatements and management turnover. *Am. Account. Assoc.* 81 83–112. 10.2308/accr.2006.81.1.83

[B22] Deshpande S. P. (1996). Ethical climate and the link between success and ethical behavior: An empirical investigation of a non-profit organization. *J. Bus. Ethics* 15 315–320. 10.1007/BF00382957

[B23] Detert J. R. Treviño L. K. Sweitzer V. L. (2008). Moral disengagement in ethical decision making: A study of antecedents and outcomes. *J. Appl. Psychol*. 93, 374–391. 18361639 10.1037/0021-9010.93.2.374

[B24] Donmez-Turan A. (2015). The relationships among love of money, machiavellianism and unethical behavior. *Can. Soc. Sci.* 11 48–59.

[B25] Dreber A. Johannesson M. (2008). Gender differences in deception. *Econ. Lett.* 99 197–199. 10.1016/j.econlet.2007.06.027

[B26] Efendi J. Srivastava A. Swanson E. (2007). Why do corporate managers misstate financial statements? The Role of option compensation and other factors. *J. Financ. Econ.* 85 667–708. 10.1016/j.jfineco.2006.05.009

[B27] Elder G. (1986). Military times and turning points in Men’s Lives. *Dev. Psychol.* 22 233–245. 10.1037/0012-1649.22.2.233

[B28] Emmons R. A. (1987). Narcissism: Theory and measurement. *J. Pers. Soc. Psychol.* 52 11–17. 10.1037/0022-3514.52.1.11 3820065

[B29] Fei T. Yu X. Gu X. (2016). Product market competition and listed company violation. *Account. Res.* 9 32–40. 35668972

[B30] Franke V. (2001). Generation X and the military: A comparison of attitudes and values between west point cadets and college students. *J. Polit. Mil. Sociol.* 29 92–119.

[B31] Gilligan C. (1977). In a different voice: Women’s conceptions of self and of morality. *Harv. Educ. Rev.* 47 481–517. 10.17763/haer.47.4.g6167429416hg5l0

[B32] Gowan M. A. Lepak D. (2007). Current and future value of human capital: Predictors of reemployment compensation following a job loss. *J. Employ. Counsel.* 44 135–144. 10.1002/j.2161-1920.2007.tb00032.x

[B33] Grant R. M. Visconti M. (2006). The strategic background to corporate accounting scandals. *Long Range Plann.* 39 361–383. 10.1016/j.lrp.2006.09.003

[B34] Griffin J. A. (2003). Challenge for the telecom industry: Converging enterprise portals and business intelligence to produce a collaborative business platform. *Thomson Media* 13:40.

[B35] Hambrick D. C. Mason P. A. (1984). Upper echelons: The organization as a reflection of its top managers. *Acad. Manag. Rev.* 9 193–206. 10.2307/258434

[B36] Hass L. Tarsalewska M. Zhan F. (2016). Equity incentives and corporate fraud in China. *J. Bus. Ethics* 138 723–742. 10.1007/s10551-015-2774-2

[B37] He H. Yuan Y. Peng J. (2012). The impact of performance appraisal orientation on employee creativity: Moderating role of employee fairness perceptions of performance appraisal. *Stud. Sci. Sci.* 30 739–747.

[B38] He X. Deng H. Wu S. Liang P. (2015). Catch-up pressure and Unethical behavior of companies: Data analysis from Chinese listed companies. *Manag. World* 9 104–124.

[B39] Hegarty W. H. Sims H. P. (1978). Some determinants of unethical decision behavior: An experiment. *J. Appl. Psychol.* 63 451–457. 10.1037/0021-9010.63.4.451

[B40] Henisz W. J. Delios A. (2003). Political hazards, experience, and sequential entry strategies: The international expansion of Japanese firms, 1980-1998. *Strateg. Manag. J.* 24 1153–1164. 10.1002/smj.355

[B41] Huang H. (2013). Financial risk prevention of moral and ethical considerations. *Res. Econ. Manag.* 12 119–123.

[B42] Jiang F. Kim K. A. (2015). Corporate governance in China: A modern perspective. *J. Corp. Finance* 32 190–216. 10.1016/j.jcorpfin.2014.10.010

[B43] Jin H. Qian Y. Weingast B. R. (2005). Regional decentralization and fiscal incentives: Federalism, Chinese style. *J. Public Econ.* 89 1719–1742. 10.1016/j.jpubeco.2004.11.008

[B44] Jones M. V. Coviello N. Tang Y. K. (2011). International entrepreneurship research (1989-2009): A domain ontology and thematic analysis. *J. Bus. Ventur.* 26 632–659. 10.1016/j.jbusvent.2011.04.001

[B45] Jones T. M. (1991). Ethical decision making by individuals in organizations: An issue-contingent model. *Acad. Manag. Rev.* 16 366–395. 10.2307/258867

[B46] Jun Z. (2017). Institutional context and firm misbehavior: An empirical research based on cross-national panel data. *Foreign Econ. Manag.* 39 114–128.

[B47] Kaptein M. (2008). *Developing a measure of unethical behavior in the workplace: A stakeholder perspective.* Thousand Oaks, CA: Sage Publications, 978. 10.1177/0149206308318614

[B48] Karpoff J. M. Scott Lee D. Martin G. S. (2008). The consequences to managers for financial misrepresentation. *J. Financ. Econ.* 88 193–215. 10.1016/j.jfineco.2007.06.003

[B49] Kish-Gephart J. J. Harrison D. A. Trevino L. K. (2010). *Bad apples, bad cases, and bad barrels: Meta-analytic evidence about sources of unethical decisions at work.* Washington, DC: Apa American Psychological Association, 1. 10.1037/a0017103 20085404

[B50] Knight J. (2002). Sexual stereotypes. *Nature* 415 254–256. 10.1038/415254a 11796975

[B51] Lant T. K. (1992). Aspiration level adaptation: An empirical exploration. *Manag. Sci.* 38 623–644. 10.1287/mnsc.38.5.623 19642375

[B52] Li W. A. Li B. (2008). An empirical study on the effect of institutional investors participating in corporate governance: Based on the data of 2004-2006 CCGINK. *Nankai Bus. Rev.* 11 4–14.

[B53] Li X. Chen B. (2013). Explosive enterprise immoral actions and ineffective government regulations: Institutional analysis of product safety and regulation. *Econ. Res. J.* 48 98–111+123.

[B54] Lian Y. Liu Y. Gao H. Luo K. (2019a). Does the improvement of the governance mechanism play a repairing role? A study based on the relationship between immoral irregularities and organizational performance. *Foreign Econ. Manag.* 41 72–84.

[B55] Lian Y. Ye W. Liu Y. (2019b). Industrial competition aspiration and organization strategic deviation: An empirical study in China. *Manag. World* 35 155–172+191–192.

[B56] Liu Y. Liu K. (2014). New trends in the study of ethical decision making in western enterprises. *Stud. Ethics* 1, 65–70.

[B57] Marcus A. A. Goodman R. S. (1991). Victims and shareholders: The dilemmas of presenting corporate policy during a crisis. *Acad. Manag. J.* 34 281–305. 10.2307/256443

[B58] McMahon J. M. Harvey R. J. (2007). The effect of moral intensity on ethical judgment. *J. Bus. Ethics* 72 335–357. 10.1007/s10551-006-9174-6

[B59] Meng Q. Li X. Cai X. (2018). Does corporate strategy influence corporate frauds. *Nankai Bus. Rev.* 21 116–129+151.

[B60] Messner S. F. Rosenfeld R. (2001). *Crime and the American dream*, 3rd Edn. Washington, DC: Bureau of Justice Statistics.

[B61] Meyer K. E. (2001). Institutions, transaction costs, and entry mode choice in Eastern Europe. *J. Int. Bus. Stud.* 32 357–367. 10.1057/palgrave.jibs.8490957

[B62] Miller Monica K. Reichert J. Flores D. (2015). “Social comparison theory,” in *The wiley blackwell encyclopedia of sociology*, ed. Ritzer G. (Oxford: Wiley Blackwell). 10.1002/9781405165518.wbeoss140.pub2

[B63] Morf C. Rhodewalt F. (2001). Unraveling the paradoxes of narcissism: A dynamic self-regulatory processing model. *Psychol. Inq.* 12 177–196. 10.1207/S15327965PLI1204_1

[B64] Nguyen Nhung T. Basuray M. T. Smith William P. Kopka D. Mcculloh D. (2008). Moral Issues and gender differences in ethical judgement using reidenbach and Robin’s (1990) multidimensional ethics scale: Implications in teaching of business ethics. *J. Bus. Ethics* 77 417–430. 10.1007/s10551-007-9357-9

[B65] O’Fallon M. J. Butterfield K. D. (2005). A review of the empirical ethical decision-making literature: 1996-2003. *J. Bus. Ethics* 59 375–413. 10.1007/s10551-005-2929-7

[B66] Peng M. W. (2000). *Controlling the foreign agent: How governments deal with multinationals in a transition economy.* Wiesbaden: Gabler Verlag, 141.

[B67] Peterson D. K. (2002). The relationship between unethical behavior and the dimensions of the ethical climate questionnaire. *J. Bus. Ethics* 41 313–326. 10.1023/A:1021243117958

[B68] Rudy B. Johnson A. (2016). Performance, aspirations, and market versus nonmarket investment. *J. Manag.* 42 936–959. 10.1177/0149206313503017

[B69] Singhapakdi A. Vitell S. J. Kraft K. L. (1996). *Moral intensity and ethical decision-making of marketing professionals.* Amsterdam: Elsevier Science Publishing Co Inc, 245. 10.1016/0148-2963(95)00155-7

[B70] Snyder H. (2019). Literature review as a research methodology: An overview and guidelines. *J. Bus. Res.* 104 333–339. 10.1016/j.jbusres.2019.07.039

[B71] Svetla M. Isabelle S. (2005). Comparing thai and US businesspeople : Perceived intensity of unethical marketing practices, corporate ethical values, and perceived importance of ethics. *Int. Mark. Rev.* 22 562–577. 10.1108/02651330510624390

[B72] Tan Y. Liao J. Li J. (2011). The evolution, mechanism and intervention of managers’ unethical behavior to organizational corruption: An analysis from the perspective of psychology and society. *Manag. World* 12 68–77.

[B73] Tan Y. Liao J. Wang S. (2012). Review and prospect of non-ethical behavior research in workplace. *Foreign Econ. Manag.* 34 40–48.

[B74] Tenbrunsel A. Smith-Crowe K. (2008). Ethical decision making: Where we’ve been and where we’re going. *Acad. Manag. Ann.* 2 545–607. 10.5465/19416520802211677 33567323

[B75] Treviño L. K. Den Nieuwenboer N. A. Kish-Gephart J. J. (2014). (Un)ethical behavior in organizations. *Ann. Rev. Psychol.* 65 635–660. 10.1146/annurev-psych-113011-143745 23834354

[B76] Valentine S. Silver L. (2001). Assessing the dimensionality of the Singhapakdi, Vitell, and Kraft measure of moral intensity. *Psychol. Rep.* 88 291–294. 10.2466/pr0.2001.88.1.291 11293045

[B77] Vardi Y. (2001). The effects of organizational and ethical climates on misconduct at work. *J. Bus. Ethics* 29 325–337. 10.1023/A:1010710022834

[B78] Walls J. Berrone P. Phan P. H. Johns H. (2012). Corporate governance and environmental performance: Is there really a link? *Strateg. Manag. J.* 33 885–913. 10.1002/smj.1952

[B79] Walsh J. P. Seward J. K. (1990). On the efficiency of internal and external corporate control mechanisms. *Acad. Manag. Rev.* 15 421–458. 10.2307/258017

[B80] Wan H. Chen X. (2010). Governance environment, rent-seeking and transaction cost: Evidence from the non-productive expenditures of Chinese firms. *China Econ. Q.* 9 553–570.

[B81] Wang Y. Zhao S. (2007). A review of the research on corporate managers’ ethics abroad. *Foreign Econ. Manag*. 03, 25–32.

[B82] Wei Y. Yi L. Longwei W. Ting L. (2012). Study on the unethical behavior: A literature review. *Manag. Rev.* 24 145–153+159.

[B83] Weihua C. (2011). Corporate scandals, reputation mechanism and management turnover. *Bus. Manag. J.* 33 38–43.

[B84] Wen W. Zhang X. Song J. (2019). Can Scholar-type CEOs curb corporate tax avoidance. *J. Shanxi Univ. Finance Econ.* 41 110–124.

[B85] Wu H. Liu H. (2006). A review of western ethical decision-making research. *Foreign Econ. Manag.* 12 48–55.

[B86] Wu J. Lebreton J. M. (2011). Reconsidering the dispositional basis of counterproductive work behavior: The role of aberrant personality. *Pers. Psychol.* 64 593–626. 10.1111/j.1744-6570.2011.01220.x

[B87] Xuewen K. Fan W. (2021). Corporate governance, earnings manipulation and abnormal CEO change. *J. Nanchang Univ. Human. Soc. Sci.* 52 55–66.

[B88] Yang J. Ming X. (2017). The process and antecedents of team ethical decision-making: A study in Chinese context. *Adv. Psychol. Sci.* 25 542–552. 10.3724/SP.J.1042.2017.00542

[B89] Yiu D. W. Hoskisson R. E. Bruton G. D. Lu Y. (2014). Dueling institutional logics and the effect on strategic entrepreneurship in Chinese business groups. *Strateg. Entrep. J.* 8 195–213. 10.1002/sej.1177

[B90] Zeming Y. Peilin W. Yuyuan F. (2020). Has the academic experience of executives affected the R&D manipulation of enterprises? *Foreign Econ. Manag.* 42 109–122.

[B91] Zhang L. Ren S. Chen X. Li D. Yin D. (2020). CEO hubris and firm pollution: State and market contingencies in a transitional economy. *J. Bus. Ethics* 161 459–478. 10.1007/s10551-018-3987-y

[B92] Zhang S. Zhang Z. Chen X. (2016). Antecedents, consequences and control of corporate corruptions: An international perspective and the Chinese path. *Q. J. Manag.* 1 111–134+138.

[B93] Zhou K. Yim C. Tse D. (2005). The effects of strategic orientations on technology- and market-based breakthrough innovations. *J. Mark.* 69 42–60. 10.1509/jmkg.69.2.42.60756 11670861

